# Ownership and use of long-lasting insecticidal nets for malaria prevention in Butajira area, south-central Ethiopia: complex samples data analysis

**DOI:** 10.1186/1471-2458-14-99

**Published:** 2014-01-31

**Authors:** Adugna Woyessa, Wakgari Deressa, Ahmed Ali, Bernt Lindtjørn

**Affiliations:** 1 Ethiopian Public Health Institute, P.O. Box 1242, Gulelle Sub-City, Addis Ababa, Ethiopia; 2 School of Public Health, College of Health Sciences, Addis Ababa University, P. O. Box 9086, Addis Ababa, Ethiopia; 3 Centre for International Health, University of Bergen, Bergen, Norway

**Keywords:** Long-lasting insecticidal nets, Ownership, Use, Highland-fringe, Ethiopia

## Abstract

**Background:**

Despite the encroaching of endemic malaria to highland-fringe areas above 2000 meters above sea level in Ethiopia, there is limited information on ownership and use of mosquito nets for malaria prevention. Thus, this study was designed to assess long-lasting insecticidal nets (LLIN) possession and use for malaria prevention in highland-fringe of south-central Ethiopia.

**Methods:**

A multi-stage sampling technique was employed to obtain household data from randomly selected households using household head interview in October and November 2008. Household LLIN possession and use was assessed using adjusted Odds Ratio obtained from complex samples logistic regression analysis.

**Results:**

Only less than a quarter (23.1%) of 739 households interviewed owned LLINs with more differences between low (54.2%) high (3.5%) altitudes (*Χ*^2^ =253, *P* < 0.001). Higher LLIN ownership was observed in illiterate (adj.OR 35.1 [10.6-116.2]), male-headed (adj.OR 1.7 [1.051-2.89]), owning two or more beds (adj.OR 2.7 [1.6-4.6]), not doing draining/refilling of mosquito breeding sites (adj.OR 3.4 [2.1-5.5]) and absence of rivers or streams (adj.OR 6.4 [3.5-11.8]) of household variables. The presence of ≥2 LLINs hanging (adj.OR 21.0 [5.2-85.1]), owning two or more LLINs (adj.OR 4.8 [1.3-17.5]), not doing draining/refilling of mosquito breeding sites (adj.OR 4.2 [1.3-13.6]), low wealth status (adj.OR 3.55 [1.04-12.14]), and < 1 km distance from absence of rivers or streams (adj.OR 3.9 [1.2-12.1]) of households was associated with more likely use of LLIN. The LLIN ownership was low in the highlands, and most of the highland users bought the bed nets themselves.

**Conclusions:**

This study found a low household LLIN ownership and use in the highland-fringe rural area. Therefore, improving the availability and teaching effective use of LLIN combined with removal of temporary mosquito breeding places should be prioritized in highland-fringe areas.

## Background

Current malaria control strategies in Ethiopia include indoor residual spraying (IRS) and long-lasting insecticidal nets (LLINs) to prevent malaria vectors biting humans inside houses. Moreover, other interventions such as effective case management and malaria rapid diagnostic tests (RDTs) are parts of the malaria control, prevention and elimination strategies [1]. Adequate evidence has been generated regarding mosquito nets provide a substantial degree of protection against deaths and illnesses from malaria [2-6]. But, community benefits depend also on factors related to net use, house condition, poverty, and behavior of malaria vectors [7-10]. The utilization of ITN has been widely advocated following the launch of the Roll Back Malaria (RBM) in 1998, aimed at halving malaria burden by 2010 through increased LLINs coverage among vulnerable groups [11,12].

The mosquito net for malaria prevention was first introduced as a piloting scheme in the late 1990s in the northern part of Ethiopia [13]. This countrywide survey showed there was a low coverage of mosquito nets in 1999, but found high willingness of respondents to use nets in the future [13]. In 2000, an estimated 0.2% of households in areas < 2,000 meters above sea level (masl) in Ethiopia owned ITN [14]. Aggregated data from the demographic health survey 2005 showed that in areas < 2,000 masl household ITN coverage risen to 6.4% in 2005 [14], Thus, in 2004, the Ethiopian Government launched a rapid scaling-up of ITN distribution and coverage that targeted households in areas at risk of malaria, below 2,000 masl [15], and followed by intensive application of other key malaria interventions since 2005 [1]. Subsequently, the LLIN coverage reached 65.6% coverage in 2007 [16], 95.0% of nets surveyed in areas < 2,000 m were LLINs. Most nets owned were nets distributed free to beneficiaries by the Federal Ministry of Health since 2005. Thus, the LLINs were primarily blue, family size (i.e. 180 × 180 × 150 cm), rectangular PermaNet® 2.0 (Vestergaard Frandsen, Copenhagen, Denmark); 94.3% of ITNs were reportedly < 3 years old [16].

Various studies showed that malaria even reaches high altitude areas laying between 2,100 and 2,400 masl [17-21]. In fact malaria transmission intensity is low in highlands, and malaria control programme prioritize populations living in malarious areas, most often located below 2,000 masl in Ethiopia [1]. Malaria epidemics at highlands are associated to abnormal weather conditions [18,22].

A recent study in similar study area has shown the importance of malaria in the high altitudes [21], which might be partly related to the warming climate. However, there is limited information regarding LLINs use in the Ethiopian highlands, especially among population living between 2,000 and 2,500 masl. These areas have been susceptible to repeated epidemic phenomenon of different magnitudes [18,22]. Such information can be important to improve planning malaria control program and selection of appropriate interventions in these areas. The objectives of this study were to assess ownership and use of LLINs in rural Butajira area in the south-central Ethiopian highlands.

## Methods

### Study area and population

This study was conducted in six kebeles (or the smallest administrative units) in the Butajira area, which is located about 130 km south of Addis Ababa. Those kebeles are parts of Butajira Rural Health Program Demographic Surveillance Site [23]. Administratively, the study was done South Nations Nationalities People Regional State and Meskan and Mareko Districts. The study area is part of an altitudinal transect, which is situated between 1,800 and 2,300 masl. Malaria is endemic with the highest transmission towards the lowlands as described in our previous work [21]. There were 58,335 people living in the Butajira DSS in 2008. Half (50.1%, n = 29,243) of the population were females. Of the total population, 46% (n = 26,834) of the people lived in our study areas. Malaria is one of the important causes of sicknesses in Butajira area. Above one-thirds (32.3%, 19,923 of 61,654) of malaria suspected people consulted out-patient department of Butajira and Enseno Health Centres, and Butajira Hospital were found microscopically confirmed malaria cases between 2004 September and 2010 August [24].

### Sample size calculation

Household dataset of malaria prevalence study was used for the LLIN ownership and use study. Thus, the sample size determined to estimate malaria prevalence (n = 750) in the present study area [21], was believed adequate for this study. Since part of the present study areas was located above 2,000 m and not covered with LLIN distribution, it was assumed that difference in LLIN ownership differ between the two areas.

### Study design and sampling procedure

**Figure 1:**
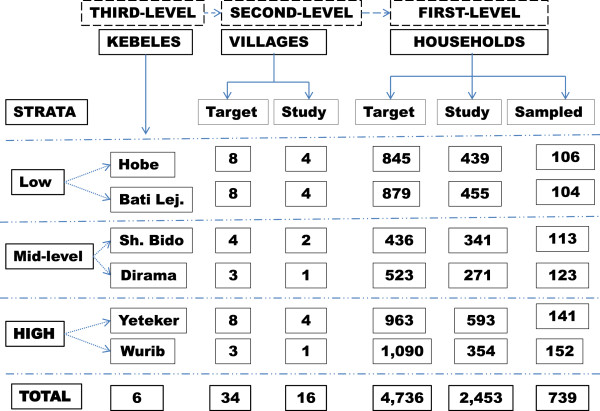
Framework of the study design.

A multi-stage sampling technique was employed to obtain data from a cross-sectional household survey. This sampling involved three stages such as kebeles, villages and households. Six kebeles (Hobe, Bati Lejano, Dirama, Shershera Bido, Yeteker and Wurib) were randomly sampled from a total of ten, which are part of demographic surveillance site in Butajira area. Those kebeles were stratified into low, mid-level, and high altitudes. The 750 households were then sampled randomly from the six kebeles and recruited from 16 villages, using probability proportion to size sampling methods (Figure 1).

### Household survey data

This is a cross-sectional survey that we did from 21st October to 6th November 2008. Household heads or their representatives were interviewed on socio-demographic and on household asset. Information on the possession of nets and its condition was collected using interview and observation. Data collectors estimated and recorded the distance of a nearby stream or river from each study household. This study considered a river or stream that is available throughout the year as permanent water body. This study considered LLIN use as reportedly at least a person has slept under a net the night prior to the survey. Hand-held Global Positioning System (Garmin eTrex®, USA) was used to measure altitudinal location of households. The altitude readings were recorded with a 7-9 m precision. This study is complied with Standards for Reporting of Diagnostic Accuracy guidelines (STARD) to improve the quality of reporting [25].

### Data management and statistical analysis

The principal investigator and an experienced research assistant from the Butajira DSS did the daily field supervision and cross-checking of the completed questionnaire. Data entry and cleaning were conducted using Epi Info version 6 (CDC, Atlanta, USA). The PASW (Predictive Analytics Software) version 20.0 statistical package (IBM Corp, Armonk, NY, USA) was used for data analysis. Descriptive statistics were performed to analyse the characteristics of the sample. Bivariate and multivariate logistic regression analysis were carried out to identify predictors of the ITNs ownership and usage. Only a few households possessed ITN in mid-level stratum, and consequently aggregated with the low altitude stratum (or < 2000 m), simply for the convenience of data analysis. Planning of antimalarial interventions has been targeted to geographical areas below 2,000 m, except in epidemic situations. Variables in the bivariate were selected for the multivariate based on a *prior* knowledge. Then, those with significance value in the bivariate were included in the multivariate. Otherwise household gender was maintained in the final model for ITN ownership. A *P* < 0.05 was considered statistically significant.

Household wealth index was computed using Principal Component Analysis (PCA) in SPSS software. Similar studies also used PCA to construct a relative household wealth index [17,26,27]. Ownership of household assets, type of usual water sources, type of product, and house construction material were used to build the wealth index as input to PCA. Factorability of the data set was checked using Kaiser-Meyer-Oklin and Bartlett’s Test of Sphericity [28]. Eleven variables with greatest weights were loaded on the first principal component and the wealth index varied from -0.256 to 13.27. Finally, all households were categorized into three categories including “lowest” ranked group (30.9%, n = 228), followed by the “middle” ranked group (35.7%, n = 264), and finally the top third in the “higher” ranked group (33.4%, n = 247).

### Complex samples data analysis

This study used data obtained through complex sample. This sampling utilized cluster sampling of kebeles, villages and households. Thus, households within clusters are more similar than households randomly sampled from the population as a whole. This sampling design effectively reduces the information contained in each degree of freedom, which is called design effects [29]. Complex sample violates the assumptions of independence of observations compelling to correct for design effects during data analysis. A study showed that data analysis that do not consider correcting for design effects leads to underestimation of standard errors and resulting to significance tests that are inappropriately sensitive [30].

A common practice to account for appropriate modelling of the complex sample is computing weights for each individual in the data set as described [31]. Briefly, weighting is used to correct disproportional sample sizes and adjust the collected data to represent the population from which the sample was drawn. The weight a case has is usually a function of the likelihood of inclusion in the sample. To adjust such distortion within a sample, every case will be assigned a weighting factor, by which the corresponding data is multiplied. This factor is determined by the proportion of the respective group or stratum in the population divided by the proportion of that group or stratum in the sample (the inverse of the sample fraction in each group).

In order to obtain a weighting factor for each case (or household), we used a formula: weighting factor equals % in population divided by % in sample. The weighting factor for each sample household was obtained. The calculated values were assigned to each sample and saved as a new variable using SPSS syntax. The other main important step in accounting for design effects is adjusting or normalizing weights. Weights were adjusted by dividing the weight by the mean of weights. Similarly, the adjusted weighting were saved to the dataset for each case and accounted for all statistic operations. Moreover, the association of predictor variables and outcome was estimated using unadjusted and adjusted odds ratio obtained from Complex Samples Logistic Regression Model.

### Ethical considerations

The study obtained ethical clearance from the Institutional Review Board of the College of Health Sciences of the Addis Ababa University, and from the Ministry of Science and Technology of Ethiopia. A written consent is obtained from the South Nations Nationalities People Regional State and Meskan and Mareko Districts. Individual informed consent was obtained from all household heads or their representatives. The necessary health education was given to the households on malaria control and prevention as well as personal protection after interview.

## Results

### Characteristics of respondents

This study used data from interview of 739 households. The response rate was 95% (739 of 750 households). Above half of the respondents (54.9%, 406 of 739) were females. The age range of participants was 18-99 years, with a mean (±SD) age of 39.2 (±14.9) years. Most of the households were male-headed (70.4%). The average family size *per* household was 5.14; family members slept in the house *prior* night to the survey were slightly lower than the actual family size (5.07) (data not shown). Table 1 shows the household and socio-demographic characteristic of study participants.

**Table 1:** Characteristics of households at low and high altitudinal strata in Butajira area, Ethiopia, October-November 2008

**Household characteristics**	**Altitudinal strata**			**Pearson chi-square; *P*-value**
	**Low (n = 286)**	**High (n = 453)**	**Total (N = 739)**	
**Respondent gender**				1.4; 0.2
Male	121 (42.3)	212 (46.8)	333 (45.1)	
Female	165 (57.7)	241 (53.2)	406 (54.9)	
**Household-head gender**				0.2; 0.6
Male	192 (67.1)	312 (68.9)	504 (68.2)	
Female	94 (32.9)	141 (31.1)	235 (31.8)	
**Education status**				49.4; < 0.001
No schooling at all	3 2 (11.2)	1 (0.2)	33 (4.5)	
Schooling*	254 (88.8)	452 (99.8)	706 (95.5)	
**Wealth status**				17.3; < 0.001
Low	107 (37.4)	121 (26.7)	228 (30.9)	
Middle	77 (26.9)	187 (41.3)	264 (35.7)	
Higher	102 (35.7)	145 (32.0)	247 (33.4)	
**Number of beds**				9.5; < 0.002
1	206 (72.0)	370 (81.7)	576 (77.9)	
2-5	80 (28.0)	83 (18.3)	163 (22.1)	
**Mosquito source reduction**				78.9; < 0.001
No	207 (72.4)	176 (38.9)	383 (51.8)	
Yes	79 (27.6)	277 (61.1)	356 (48.2)	
**Permanent water body**				177.6; < 0.001
None	106 (37.1)	5 (1.1)	111 (15.0)	
< 1 km	96 (33.6)	236 (52.1)	332 (44.9)	
≥1 km	84 (29.4)	212 (46.8)	296 (40.1)	
**Occupation farming**				9.2; 002
No	96 (33.6)	203 (44.8)	299 (40.5)	
Yes	190 (66.4)	250 (55.2)	440 (59.5)	

*Read and write to complete grade 12.

### LLINs possession and use

Below a quarter (23.1%, n = 171) of the 739 households surveyed, owned at least an LLIN, with a mean of 1.54 [95% CI 1.45-1.63] LLINs *per* household. Household LLIN ownership was higher in low altitude (n = 155) than in high altitude (n = 16) (Table 2). Below half (47.4%) of the households possessing ≥2 LLINs in which all of them were from low altitude.

**Table 2:** Relationships of household characteristics of and ITN ownership status in Butajira area, Ethiopia, October-November 2008

**Household characteristics**	**Household own LLINs**			**Pearson chi-square; *P*-value**
	**No (n = 568)**	**Yes (n = 171)**	**Total (N = 739)**	
**Respondent gender**				0.78; 0.4
Male	261 (46.0)	72 (42.1)	333 (45.1)	
Female	307 (54.0)	99 (57.9)	406 (54.9)	
**Household-head gender**				3.8; 0.05
Male	377 (66.4)	127 (74.3)	504 (68.2)	
Female	191 (33.6)	44 (25.7)	235 (31.8)	
**Education status**				89.2; < 0.001
No schooling at all	3 (0.5)	30 (17.5)	33 (4.5)	
Schooling	565 (99.5)	141 (82.5)	706 (95.5)	
**Wealth status**				21.7; < 0.001
Low	153 (26.9)	75 (43.9)	228 (30.9)	
Middle	224 (39.4)	40 (23.4)	264 (35.7)	
Higher	191 (33.6)	56 (32.7)	247 (33.4)	
**Number of beds**				13.2; < 0.001
1	460 (81.0)	116 (67.8)	576 (77.9)	
2-5	108 (19.0)	55 (32.2)	163 (22.1)	
Mosquito source reduction*				60.0; < 0.001
No	250 (44.0)	133 (77.8)	383 (51.8)	
Yes	318 (56.0)	38 (22.2)	356 (48.2)	
**Distance of permanent water body**				76.6; < 0.001
No permanent water body	51 (9.0)	60 (35.1)	111 (15.0)	
< 1 km	260 (45.8)	72 (42.1)	332 (44.9)	
≥1 km	257 (45.2)	39 (22.8)	296 (40.1)	
**Occupation farming**				5.5; 02
No	243 (42.8)	56 (32.7)	299 (40.5)	
Yes	325 (57.2)	115 (67.3)	440 (59.5)	
Altitudinal Strata				253.0; < 0.001
Low	131 (23.1)	155 (90.6)	286 (38.7)	
High	437 (76.9)	16 (9.4)	453 (61.3)	

*Refilling & draining of mosquito breeding sites.

Among net-owning households, 83.0% (142 of 171) had a family member who slept under a net the night *prior* to the survey. Of the households reportedly use their nets 76.1% (108 of 142) had nets with holes. LLINs with holes showed differences between households located at low (78.5%, n = 106) and high (28.6%, n = 2) altitudes (*Χ*^2^ =9.1, *P* = 0.003).

### Main reasons for non-possession of LLIN

More than three-fourths (76.9%, n = 568) of the households did not have any LLIN during the survey (Table 1). A few of those households (13.2%, n = 75) had net previously. Main reasons respondents cited for non-possession of LLIN during the survey were: do not know sources of LLIN and not available in the area (61.4%, n = 212), worn out and discarded (19.4%, n = 67), believed LLIN do not protect/not important (14.5%, n = 50), and shortage of money and narrow sleeping space (4.6%, n = 16) (data not shown).

For households reportedly using their LLIN (n = 142), the main sources of LLIN at low altitude (n = 135) were health facilities (n = 39, 28.7%), District Health Office (n = 95, 70.5%) and NGO (n = 1, 0.7%); whereas at high altitude (n = 7) health facilities (n = 3, 42.3%), market/shop (n = 3, 43.0%) and other sources (n = 1, 14.8%) (Data not shown).

### Determinants of LLINs possession and use

This study found household LLIN ownership associated with various household factors such as socio-demographic, status of practising mosquito source reduction, other breeding places instead of permanent water body and number of beds in the house (Table 3). Household heads with no formal education had above 35-fold higher LLIN ownership compared to those with formal education. Household characteristics such as absence of main water body (above 6-fold), not practising mosquito source reduction (more than 3-fold) and presence of two and more beds in the household were significantly associated with increased household LLIN possession than their counterparts. Male-headed households were also associated to increased LLIN possession than female-headed households. The households with LLIN observed hanging, two and more number of net owned, not practising source reduction and farming occupation showed statistically significant association with highly likely to use LLIN. Of those, the presence of more LLIN hanging in a household is a good predictor of net usage (Table 4).

**Table 3:** Predictors of household ITN ownership (N = 739) obtained from complex samples logistic regression model, Butajira area, Ethiopia, October-November 2008

**Household characteristics**	**Unadj. OR**	**95% CI**	**adj. OR**	**95% CI**
No schooling at all				
Schooling	**31.3**	**9.4-104.4**	**35.1**	**10.6-116.2**
Educated	1		1	
**Wealth status**				
Low	1.5	1.0-2.2	1.3	0.7-2.4
Middle	**0.5**	**0.3-0.9**	0.8	0.4-1.5
High	1			
**Head of household gender**				
Male	1.3	0.9-2.0	**1.7**	**1.05-2.89**
Female	1		1	
**Number of beds**				
1	1			
≥2	**2.1**	**1.4-3.1**	**2.7**	**1.6-4.6**
**Mosquito source reduction**				
No	**4.7**	**3.1-7.0**	**3.4**	**2.1-5.5**
Yes	**1**		**1**	
**Permanent water body**				
None	**6.5**	**3.9-10.8**	**6.4**	**3.5-11.8**
< 1 km	1.9	1.2-2.9	1.1	0.6-1.9
≥1 km	1		1	

**Table 4:** Predictors of household LLIN use (N = 171) obtained from complex samples logistic regression model, Butajira area, Ethiopia, October-November 2008

**Household factors**	**Unadj. OR**	**95% CI**	**adj. OR**	**95% CI**
**Number of LLINs hanging**				
0	1			
1-2	**12.1**	**3.5-42.4**	**21.0**	**5.2-85.1**
**Number of ITNs owned**				
1	**1**			
2-4	**4.3**	**1.5-12.1**	**4.8**	**1.3-17.5**
**Mosquito source reduction**				
No	**2.8**	**1.2-6.9**	**4.2**	**1.3-13.6**
Yes	1		1	
**Wealth status**				
Low	**3.3**	**1.1-9.9**	**3.55**	**1.04-12.14**
Middle	0.7	0.3-1.9	0.51	0.15-1.69
High	1		1	
**Permanent water body**				
**No permanent water body**	1.3	0.5-3.3	1.7	0.6-5.4
< 1 km	2.1	0.7-5.8	**3.9**	**1.2-12.1**
≥1 km	1		1	

## Discussion

The present study showed that LLIN use was highest for households owning more LLIN in < 2, 000 m areas, where the government had distributed bed nets to all households. LLIN possession was low in the highlands, and most of the highland users bought the bed nets themselves. Interestingly, the poor, families with low education status living in the lowlands and adjacent to potential mosquito breeding sites used bed nets more than others, probably reflecting that perceived risk of malaria is more important than wealth and educational status.

There are some limitations in the present study. This study used data obtained using a cross-sectional household survey of LLIN ownership. Because of the seasonal differences in malaria risk as found in recent study in the same study area [21,32], Consequently, the present finding of LLIN use might be biased and under estimated as malaria risk was low during 2008 compared to 2009. Household LLIN use was obtained by self-report and that may be subject to social-desirability bias. This might be resulting in an overestimation of LLIN usage as a previous study reported [33]. Secondly, this study was a cross-sectional survey done during a peak malaria transmission season, and we might thus have overestimated the real estimate of net usage. A previous study has shown seasonal differences in net utilization in highland areas [34]. The data analysis considered complex samples into account and avoided reporting biased information. Despite these limitations, this study has shown some strengths to draw valuable conclusions that might help in improving designing malaria interventions at highland-fringe areas. This study tried to explicitly examine the use of LLINs by all household members, which makes it is different from previous studies that focused only on the vulnerable groups. The populations at high altitude are presumably at increased risk of epidemics without suitable interventions.

The low LLIN ownership observed is similar to a previous study [35], and also comparable to another study [9]. The LLIN use in this study was the highest compared with the Ethiopian 2007 MIS [16] while similar in a highland area of Kenya [36]. The present finding of LLIN ownership in areas >2,000 masl, which is beyond the altitude limit for LLIN distribution and scaled-up interventions, is in agreement with the recent finding of country wide survey in 2011 [37]. It was found that net use is associated with malaria exposure, and low net use in highland areas with low malaria transmission might not be surprising, though the bed net distribution mechanisms differed.

The present finding of increased net possession in illiterate household heads is in contrast with an earlier study in Ethiopia [38]. The finding related to educational status and LLIN ownership can be more explained by a recent study that demonstrated knowledge in malaria prevention and control might not result from formal education only, but also other sources such as non-formal and informal education [39]. The present study showed that households with additional beds had increased net possession. Obviously, more beds are expected in households with a larger family, and allocate additional nets during the distribution as *per* the national LLIN distribution strategy [15]. A positive association of household family size and LLIN ownership was documented [36]. The negative association between absence of rivers or streams adjacent to households and LLIN ownership found in the present study is in accordance with a previous study [38]. Following the cessation of main rainy season the creation of transient water pools appears common in areas of flat terrain. This ecological setting has been considered as suitable for breeding of *Anopheles arabiensis,* the main malaria vector in Ethiopia, [17,40]. Similarly, *Anopheles arabiensis* has been recognized as the predominant species that entails malaria transmission in highlands of Ethiopia [18,22], and in the highlands of Kenya [34]. Moreover, an increased net possession in male-headed households was in agreement with a study in Nigeria [41].

The current finding of households with at least an LLIN seen hanging were more likely to use their net is in harmony with other studies [9,42,43]. A few of the households surveyed had hanging nets during the survey. A study from Zambia also demonstrated that the strongest factor influencing net utilization was the presence of an LLIN hanging [43]. They reported of those LLINs hanging; only 10% were not used the previous night. The present finding of about five-fold highly likely to use net in households owning two or more LLINs is in harmony with studies in Ethiopia [9,44], and various African countries [36,45]. LLIN use increase as more number of nets is available within LLIN-owning households. The present result showing increased LLIN use among households not using removal of mosquito breeding places using refilling and draining is in line with another study [46]. This suggesting other malaria prevention activities/products appeared to substitute for nets rather than complements. The finding of higher net use in households living adjacent to rivers or streams than their counterparts is in agreement with a study [47]. Thus, households with increased risk of getting malaria are more aware of the increased risk and more inclined to use protective measures. Households in the low wealth category had higher LLIN use as documented by other studies [46,48].

Since 2007, the Ethiopian malaria control strategy is targeted to achieve 100% coverage of all households at risk of malaria, < 2000 masl. Instead, we observed that above a quarter of the households geographically located between 1,900 masl and 1,999 masl were not covered by net distribution. But the parasitological survey demonstrated the presence of malaria infection throughout those areas [21]. Households located at high altitude, presumably at low malaria risk, got their nets from different sources than those living at low altitude that were obtained through government channels. More interestingly, the ownership of LLIN by more than a third of the households located in areas above 2,000 m as demonstrated in the MIS 2011 [37], which might imply the importance of improving LLIN distribution that has been limited to areas < 2,000 m.

Presumably, nets with holes owned by poor families were failed to protect man-vector contact and malaria infection. A current study revealed higher proportion of LLINs in poor condition as also found in the MIS 2011 [37]. Increased malaria infection was found in areas < 2,000 masl [32]. Probably, damaged LLIN and low coverage could partly be a failure in ensuring protection of malaria in the present study area. A study found individuals from the most poor households were more likely to sleep under nets with holes compared to the least poor [49]. In order to obtain full protection from malaria infection using LLIN requires possessing an intact net and persistently sleeping under net every night.

## Conclusion

In conclusion, this study shows there is a deficit between the nationally targeted household LLIN ownership and use in the highland-fringe area of south-central Ethiopia. Therefore, malaria interventions should focus on improving the availability and teaching effective LLIN use combined with removal of temporary mosquito breeding places in the populations at risk of highland-fringe areas. Future LLIN ownership and use studies should emphasise on concurrent investigation of individual parasitological status and LLIN condition.

## Competing interests

The authors declare that they have no competing interests.

## Authors’ contribution

AW contributed in conception and design, acquisition of data, analysis and interpretation of data, and drafting the manuscript. WD substantially contributed to conception and design of the study and substantially revisiting the drafted paper. AA substantially contributed to conception and design of the study, reviewing the manuscript and revisiting it critically for important intellectual content. BL substantially contributed to conception and design, analysis and interpretation of data and reviewing the manuscript. BL, AA, WD and AW reviewed the paper and all authors approved the final version.

## Pre-publication history

The pre-publication history for this paper can be accessed here:

http://www.biomedcentral.com/1471-2458/14/99/prepub
